# A randomised crossover comparison of two endotracheal tube introducers: the FROVA and the Flexible Tip Bougie for GlideScope intubation of a difficult airway manikin by infrequent intubators

**DOI:** 10.1186/s12245-020-00298-6

**Published:** 2020-07-17

**Authors:** John Cormack, Bridget Langley, Louisa-Rose Bhanabhai, Roman Kluger

**Affiliations:** 1 The Faculty of Medicine, Dentistry and Health Sciences, University of Melbourne, Building 181, Grattan St, Melbourne, 3010 Australia; 2 Department of Anaesthesia and Acute Pain Medicine, St Vincent’s Hospital Melbourne, 41 Victoria Parade, PO Box 2900, Fitzroy, VIC 3065 Australia

**Keywords:** Intratracheal intubation, Manikin, Teaching materials, Airway management

## Abstract

**Background:**

This unblinded randomised crossover study compares two endotracheal tube introducers (ETIs): the FROVA and the “Flexible Tip Bougie” (FTB), in an airway manikin mimicking difficult intubation with a percentage of glottic opening view of 30%. Participants were Emergency Medicine and Anaesthesia trainees with recent experience of less than twenty patient intubations. The primary outcome was time to intubation, further divided into time taken to pass the ETI and time to railroad the endotracheal tube (ETT) over the ETI. The secondary outcome was the difficulty of intubation.

**Results:**

The median total time to ETT placement was significantly shorter with the FTB (37.5 s) compared with the FROVA ETI (63.0 s), *P* = 0.0006. The median difficulty reported (scores 0–10 with 0 being no difficulty) with the FTB was 2 compared with 5 for the FROVA, *P* < 0.0001.

**Conclusions:**

The FTB enabled significantly faster and easier placement of the endotracheal tube compared with the FROVA in inexperienced hands intubating a difficult intubation manikin.

## Introduction

### Background

Intubation is challenging for the novice intubator in the emergency department where suboptimal positioning, urgency and injuries can add to the difficulty. The GlideScope (Verathon, Bothell, WA) has given novices an improved tool compared with traditional Macintosh blade direct laryngoscopy [1–3], allowing a rapid learning curve, less cervical spine movement and fewer oesophageal intubations [4]. The problem of anterior tracheal impingement, causing the “can see but can’t intubate” phenomenon, is a common issue and while most intubations are eventually achieved; this may involve multiple attempts with the possibility of laryngeal trauma or hypoxia.

Devices to assist GlideScope intubation include stylets and endotracheal tube introducers (ETIs) (bougies), and the latter have been used to improve intubation success when difficulty is encountered [5]. These static guides can be bent into a curve which cannot be altered during each intubation attempt. Alternatively, some ETIs have a flexible tip replicating the action of a flexible bronchoscope (fiberscope) tip. When a fiberscope is used by experienced intubators as a “dynamic introducer”, it is superior to a stylet in difficult GlideScope intubation [6].

**Fig. 1:**
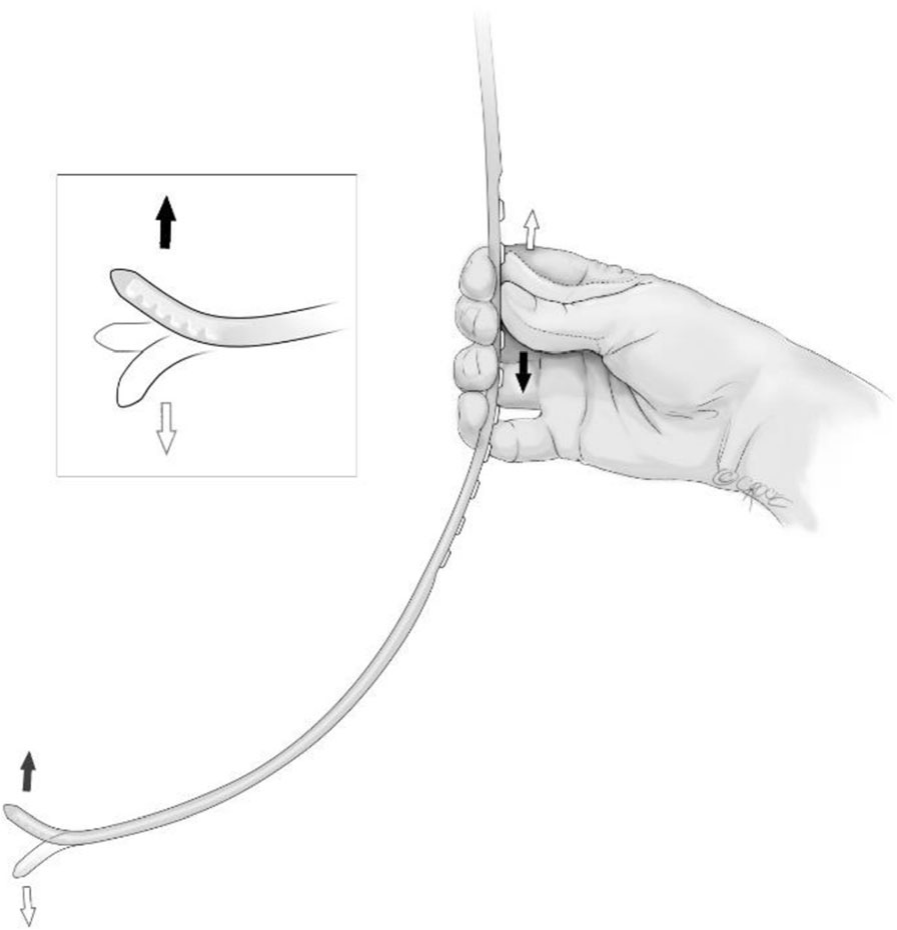
Flexible Tip Bougie. Demonstrating flexion and extension via downward (dark arrow) or upward (white arrow) pressure upon the ridged slide on the shaft. Diagram copyright, Beth Croce Bioperspective.com

A commonly used ETI is the FROVA (Cook Medical, Bloomington, IN) which can be bent into a curve matching that of the GlideScope and placed in the trachea. Once the ETI is intratracheal, it is used as a guide for the endotracheal tube (ETT) to pass over (railroad). It is hollow allowing oxygenation if needed. When space is limited or the glottic view is reduced, an ETI allows a clearer view of the tip entering the larynx due to its small diameter. The “Flexible Tip Bougie” (FTB, Construct Medical, Hawthorn, VIC) has a permanent curve matching the GlideScope and the ability to flex or retroflex at the tip using pulsion or traction on a low-profile slide on the shaft (Fig. 1). A previous difficult airway manikin study has compared these two devices with experienced intubators and found that the FTB was superior with reduced time to intubation and easier intubation [7].

### Importance

The global corona virus disease 19 (COVID-19) pandemic has created an unprecedented demand for rapid and successful endotracheal intubation, often under unfavourable conditions of hypoxia in locations including intensive care and the emergency department. The intubation may need to be performed by staff who have previously only intubated occasionally, with ideal conditions and under supervision. The study was devised prior to this global emergency; however, the aim to help define the fastest and easiest intubation method for the novice intubator under difficult circumstances is more relevant than ever.

### Goals

Our hypothesis was that Glidescope intubation aided by the FTB would be quicker and easier to use than the FROVA with inexperienced intubators.

## Methods

### Study design and setting

A single centre randomised, unblinded crossover trial approved by the institutional Ethics Board (LRR171/18).

### Selection of participants

**Fig. 2:**
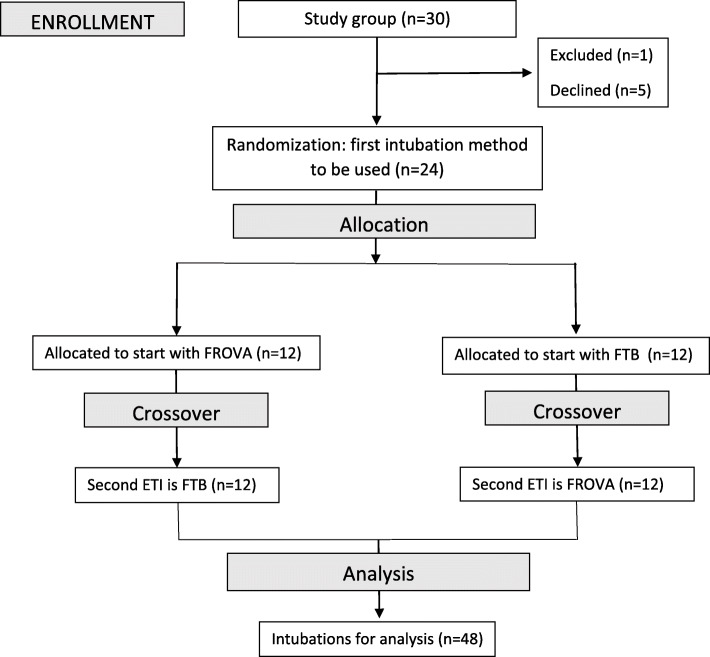
Flow chart of study protocol

Thirty novice intubators were recruited, including Emergency Medicine trainees and Anaesthetic residents. All trainees able to attend during normal working hours were invited to participate. The exclusion criterium was greater than twenty patient intubations in the previous calendar year. Five enrolled participants were unable to attend during the specified times. One participant revealed greater experience and was excluded from the analysis. Twenty-four participants completed the study and were included for analysis. A flow chart from enrolment to analysis is shown (Fig. 2).

### Interventions

Participants were consented, given instructions and shown an instructional video demonstrating both devices. The video was reviewed on the study day, and questions were invited. Each participant was given the opportunity to examine both ETIs before commencing.

**Fig. 3:**
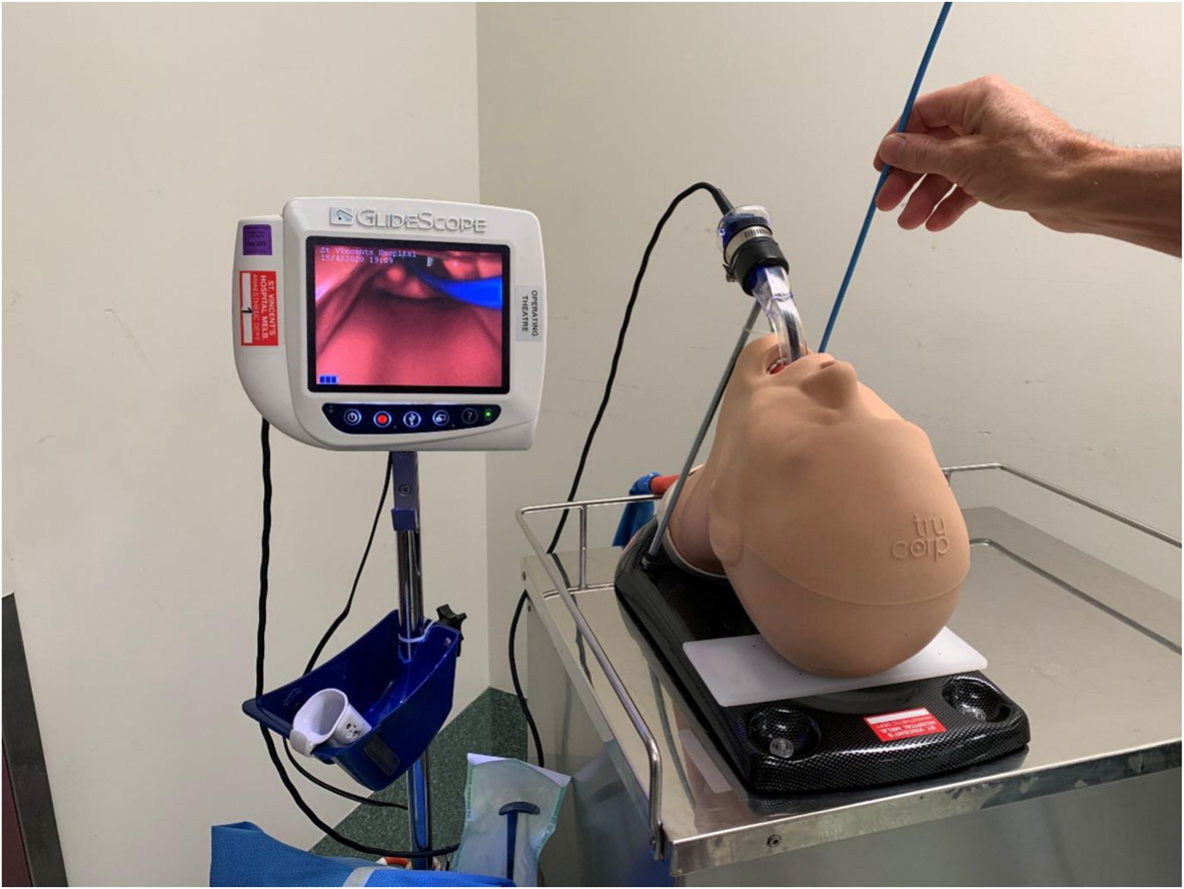
AirSim Advance X Manikin modified to extend the head and with tongue insert fully inflated. GlideScope held in static position by rods attached to the base of the manikin

The AirSim Advance X manikin (Trucorp, Lurgan, N. Ireland) was positioned in the simulation centre or in a quiet room within the operating theatre complex. The GlideScope/manikin setup offered a percentage of glottic opening (POGO) view of 30% [8], unchanged during the study period. Silicone lubricant was applied to each ETI. The GlideScope was a Cobolt AVL model with a size 4 GVL Stat hyperangulated blade (Verathon, BC, Canada) fixed into position throughout the study on a frame attached to the base of the manikin (Fig. 3). Subsequent MacIntosh laryngoscopy revealed a Cormack and Lehane view grade three [9].

Participants were randomised to either the FROVA or FTB first. Total time to intubation was the time from receiving the ETI until the ETT was placed in the mid-trachea and was further broken down into two components: the time to placement of the ETI and time from placement of ETI until placement of the ETT (Appendix: supplementary video 1). Difficulty was rated for each phase on a scale from zero to ten (ten = most difficult). Five minutes was the arbitrary point at which intubation attempts would be abandoned. Interviews were conducted at completion.

The second phase of the crossover study was a replication of the first phase using the remaining ETI type (either FROVA or FTB). The order of ETI used was randomised in permuted blocks of four. The numbers for the study were derived from a similar study looking at time to intubation comparing the same two ETIs with experienced intubators^7^.

**Additional file 1: Supplemental video 1.**

### Measurement

With the backup of audiovisual recording, an investigator stood beside the participant, passing the equipment and was responsible for determining and signalling the insertion of the ETI and then the ETT by watching the GlideScope screen. A second investigator was observing and recording times for each endpoint. A third investigator reviewed video recordings to confirm accuracy of endpoint times.

### Outcomes

Time until the ETT reached the mid-trachea was the primary outcome, broken down into two component parts: the time to insert the ETI into the mid trachea and the time to fully insert the ETT over the ETI (Supplementary video 1). Secondary outcomes were difficulty of ETI insertion and difficulty of ETT passage over the ETI. We also collected descriptive data to highlight any specific problems encountered.

Upon review of the descriptive data and audiovisual recordings, the most frequent difficulty with the FROVA was that the tip impinged upon the anterior tracheal wall (5/24) preventing further insertion to the mid trachea and making it impossible to advance the ETT beyond the impingement. This did not occur with the FTB (0/24) as the impingement was prevented by extending the tip down towards the trachea. In all cases, the time to placement of the ETI was recorded as the time to reach mid-trachea regardless of whether one or more attempts at ETT placement were made prior to this being achieved.

We measured the performance of the ETI device and eliminated the skill of visualising the larynx. Fixing the GlideScope in a static position throughout ensured a consistent view of the glottis during the study and removed a laryngoscopy learning effect favouring the second arm. Dividing the intubation time into two epochs was important to discern if the process of railroading the tube over the ETI was different between the two devices.

Paper data entry was transcribed by a research nurse onto an Excel database (Microsoft) and rechecked for accuracy against the paper record by the principle investigator. Spurious results were compared with the video and digital clock recordings.

### Analysis

Times and difficulty scores were compared between groups using Wilcoxon matched pairs tests as the data was not normally distributed. A *P* value < 0.05 was deemed significant. All statistical analyses were performed using Stata/IC 14.2 for Windows, StataCorp LLC, TX, USA.

## Results

### Characteristics of study subjects

Emergency Medicine trainees were in year one to four and Anaesthetic residents were in their second year of residency, rotating through an anaesthesia term. Many had not recorded human and manikin intubation numbers; however, strict assurance of less than 20 human intubations in the previous 12 months was required in all cases. Characteristics of the participants experience are summarised (Table 1).

**Table 1:** Participant demographic data

Mean age (years)	29.7
Mean experience (months)	22.3
Mean solo intubations	4.8
Trainee stream	
Anaesthetic residents	*n* = 7
Emergency registrars	*n* = 17
Previous FROVA use	22/24
Previous FTB use	1/24

### Main results

**Fig. 4:**
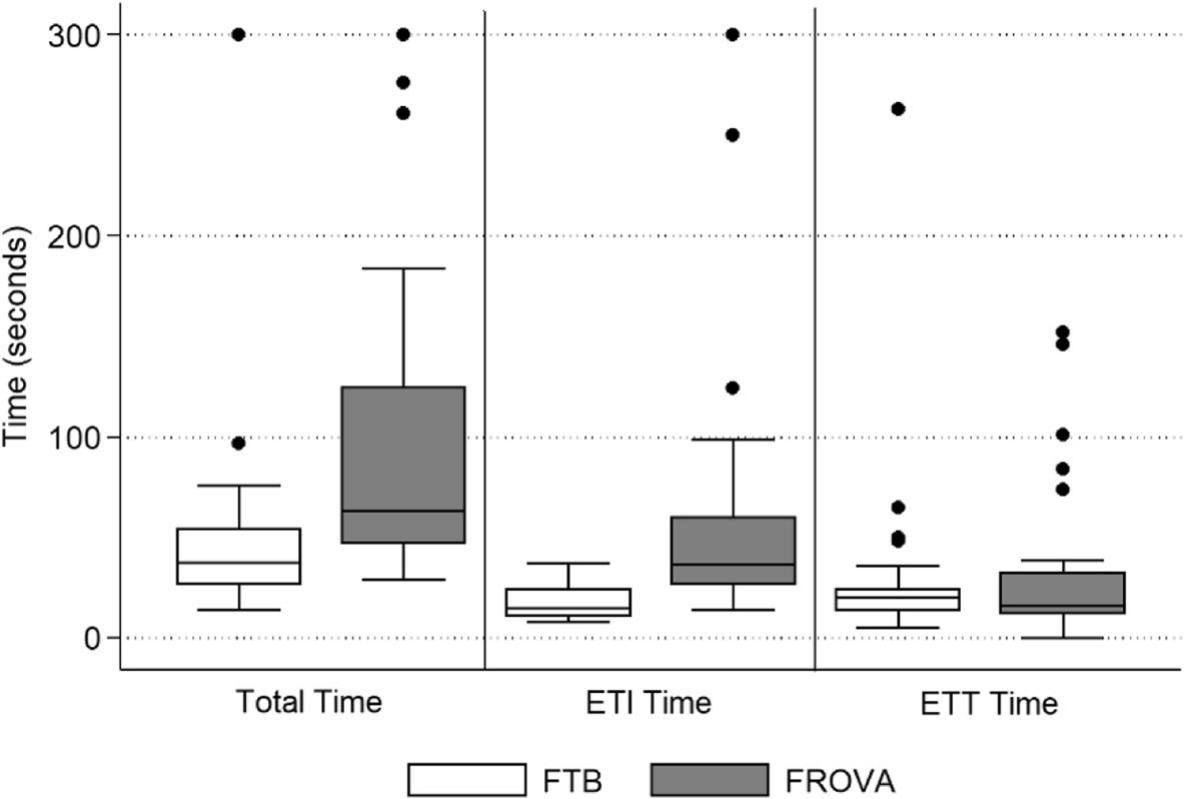
Left graph: total time from receiving the ETI until ETT fully inserted. Central graph: time until ETI reached mid trachea. Right graph: time to railroad ETT over the ETI

The median total time to endotracheal tube placement was significantly shorter with the FTB (37.5 s) compared with the FROVA ETI (63.0 s), *P* = 0.0006. One participant using the FROVA and one using the FTB were stopped at 5 min without being able to place the ETT in the mid trachea. These were both recorded as 300 s and were included in the analyses. The advantage of the FTB was due to quicker placement of the ETI rather than a difference in time to railroad the ETT. Median time to place ETI with FTB was 15 s and with FROVA 36.5 s (*P* = 0.0001). Median time from placement of ETI until ETT was within the trachea with the FTB was 20 s and with the FROVA 16 s (*P* = 0.9) (Fig. 4).

**Fig. 5:**
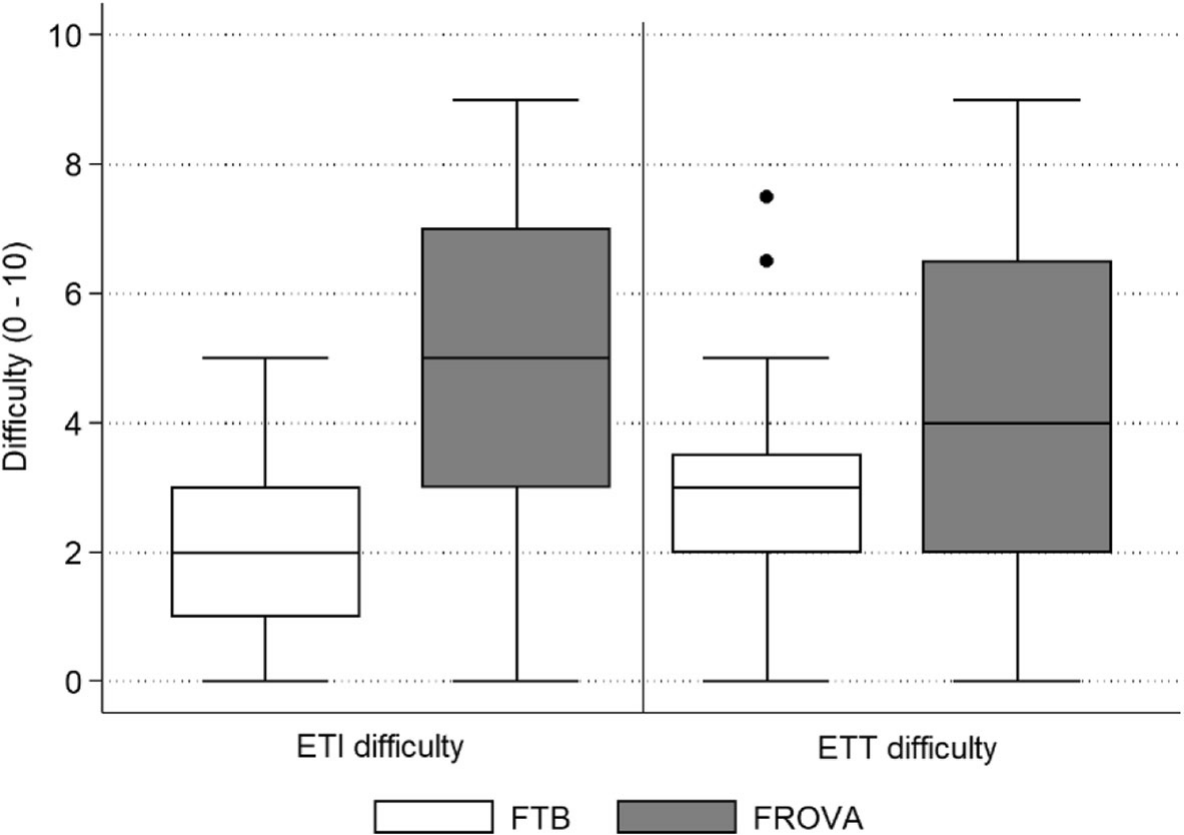
Difficulty scores (0 = very easy, 10 = very difficult). Left graph: passage of ETI. Right graph: passage of ETT over ETI

The median difficulty (0–10) of placing the ETI was significantly in favour of the FTB = 2 compared with FROVA = 5 (*P* = < 0.001). For the passage of the endotracheal tube over the ETI, the median difficulty with the FTB was 3 compared with FROVA 4 (*P* = 0.02) (Fig. 5).

The median difference in total time to intubation between the two ETIs was 25.5 s; however, this varied with which ETI was used first, 44.5 s (FROVA first) vs 15.5 s (FTB first), probably indicating a learning effect (Table 2).

**Table 2:** A learning effect favouring the second ETI used

Median times in seconds					
All participants (*n* = 24)					
**ETI type**	ETI time**	ETT time	Total time**	ETI difficulty**	ETT difficulty*
FTB	15	20	37.5	2	3
FROVA	36.5	16	63	5	4
FROVA ETI first group (*n* = 12)					
**ETI type**	ETI time**	ETT time	Total time**	ETI difficulty*	ETT difficulty*
FTB	11	18.5	29.5	2	2.5
FROVA	41	16	74	5	5
FTB ETI first group (*n* = 12)					
**ETI type**	ETI time**	ETT time	Total time	ETI difficulty*	ETT difficulty
FTB	20	20.5	38.5	2	3
FROVA	36.5	14.5	54	5	3.5

**P < 0.01; *P < 0.05

## Discussion

An advantage of the FTB compared with the FROVA both in time taken (median 37.5 vs 63 s) and ease of intubation was demonstrated. This is due to the ETI placement (median 15 vs 36.5 s), not subsequent railroading of the ETT. This advantage was reduced but still significant in the group using the FTB first. Familiarity with the FROVA device (previous FROVA use by 22/24 participants) may have been expected to contribute to improved performance with this device and may have biased the study from finding an even greater advantage with the FTB.

In a retrospective data analysis of first pass and overall intubation success, Mosier et al. studied difficulty associated with emergency department intubations revealing an 82% first pass success rate with the GlideScope [10]. Sakles et al. reported a close association between adverse events and multiple attempts at intubation in the emergency department [11]. With experience, ETIs (bougies) have been useful in MacIntosh laryngoscopy and intubation, but with the GlideScope, the bougie has not made a significant improvement [12, 13], compared with a stylet.

The GlideScope’s decreased working space and acute laryngeal entry angle compared with the MacIntosh laryngoscope has led to investigation of the “can see but can’t intubate” situation. Case reports have suggested that the flexible tip of a videobronchoscope (fiberscope) can prevent ETT impingement on the trachea [14, 15], and a prospective clinical trial showed that a flexible tip ETI (fiberscope) was superior to a preshaped stylet [6]. A comparison of the FROVA and the FTB with experienced intubators using the GlideScope showed faster and easier intubation with the FTB [7], and another manikin study using a MacIntosh laryngoscope showed superiority of the FTB during simulated cardiac arrest [16]. The FTB provides a cheaper, accessible alternative to the fiberscope, requiring only one intubator. Despite its recent introduction and a paucity of studies, the FTB is already listed on an emergency department COVID-19 intubation trolley [17].

## Limitations

Manikin studies can only approximate clinical conditions, but they are justifiable and ethical when a new device is used with novice intubators [18]. The 30% glottic view and an anterior larynx (seen at the upper border of a GildeScope screen) is reproducible with various models of manikin and adaptations to increase difficulty, as long as the laryngoscope is fixed. We designed the study so that the laryngoscopic view, the acute angle to be negotiated into the trachea and the friction encountered were reproducible and realistic.

Our “time to intubation” excludes laryngoscopy time. Sharma outlined the limitations of GlideScope “intubation” as opposed to “laryngoscopy”, suggesting that the two should be considered separately [19]. Previous comparative manikin studies include laryngoscopy time, measuring the time the blade enters the mouth until the ETT is fully inserted [12, 16, 20]. By excluding laryngoscopy and providing a static, constant view of the larynx, we provided the same view for each participant in both arms of the crossover study. Therefore, the time to ETI insertion is neither likely to be primarily due to the variable laryngoscopy skill of the intubator, nor a laryngoscopy learning effect with the second device used. The laryngoscopy variability in novices may have been greater than the difference found between the ETIs studied, decreasing the power.

The study was unblinded. Blinding would have increased complexity possibly introducing errors of recording or processing.

## Conclusions

In summary, our study examines a specific (yet common) difficult intubation problem which requires an initial sharp angulation anteriorally of the ETI or ETT into the larynx and then a redirection downwards into the trachea in order to achieve intubation. In this simulated situation, the FTB is superior to the FROVA. COVID-19 has created a global need for efficient intubation under difficult circumstances by intubators of variable experience. Historically, comparative quality analysis and then prospective clinical trials have followed case reports and manikin studies when a new device has shown an advantage worth pursuing.

## Data Availability

The data is available in paper and electronic database form and is securely stored in locked and password protected form in our hospital department (Department of Anaesthesia and Acute Pain Medicine, St. Vincent’s Hospital Melbourne). The datasets generated during and/or analysed during the current study are available from the corresponding author on reasonable request.

## Abbreviations

ETI: Endotracheal tube introducer = “Bougie”
ETT: Endotracheal tube
FTB: Flexible Tip Bougie
COVID-19: Corona virus disease 19
